# The National Pension Fund

**Published:** 1887-09-24

**Authors:** 


### The National Pension Fund.

It will save a good deal of trouble if nurses and other hospital officials who propose joining the National Pension Fund will send their names and other particulars to *the Secretary of The Hospitals Association*, Norfolk House, Norfolk Street, W.C., and not to the Editor of The Hospital. Names continue to arrive in considerable numbers, but if those interested desire the Fund to be started before the end of the Jubilee year they must influence and interest all their friends immediately.

Readers will please note that in sending in their names, nurses and other hospital officials are not committing themselves to join any particular Fund, but simply stating their willingness to join "a Pension Fund if the rules and regulations are such as they approve."

By an Infirmary Matron.

I am glad to have the opportunity of saying how entirely I approve the idea of a Pension Fund for Nurses. I beg to enclose my name, with those of several of my nurses. I quite agree with some of your correspondents that the general feeling, though greatly in favour of the scheme, is strongly opposed to the notion of joining any society where the money paid would not be returnable at marriage or death.

By a Nurse.

Will you kindly put my name on the register for trained nurses as supporter of a provident Pension and Sick Fund for hospital officials and nurses? If carried to a successful issue, this fund will meet a want that has long been felt. We cannot be too grateful to Her Majesty for her generous gift, and the kind Christian feeling which dictated it.

### WANTED, A HOME FOR AN INCURABLE EPILEPTIC.

Is there any home or institution of any kind that will take in the following case:—George Dowdon, a working man, about twenty-eight years of age, fairly educated, very respectable, and having a good character, is subject to severe attacks of epilepsy, during which he is very violent. His mother is dead, and his father's wife objects to him at home. His father allows him 1s. a week, and he lives in a cottage alone, where he has often been found by neighbours unconscious, and in imminent danger from fire or injuries by his falls. People in the neighbourhood, for whom he has worked, would be willing to help towards his maintenance, could we find anyone to take charge of him, but, owing to the nature of his illness, no one will accept him as a lodger. One old couple tried to take him in, but could not manage him during the attacks. He was for some time in the Epileptic Hospital in London, but was discharged from there as incurable. At present he is in the Holborn Union; he objects to remaining in the workhouse, because he cannot there obtain the medical treatment which helps to keep off the fits, and he cannot be admitted to the workhouse infirmaries during their intervals. He is most anxious to be protected from himself, as he says he feels he is dangerous when the attacks are coming on, and he is so afraid of doing any harm to anyone. He is a native of Marple, Derbyshire. Ordinary homes for incurables will not take in epileptics.

A. P.

*** After careful inquiry, we regret to state we are unable to find any home which would receive a case of this nature (incurable epileptic), but there is a Pension Fund in connection with the National Hospital for the Paralysed and Epileptic, Queen Square, Bloomsbury, for the benefit of such cases.—[Ed. T. H.]

